# The end-world delusion with religious content, apocalyptic variant

**DOI:** 10.1192/j.eurpsy.2021.2038

**Published:** 2021-08-13

**Authors:** O. Borisova, G. Kopeyko, E. Gedevani, P. Orehova

**Affiliations:** 1 Investigation Group Of Specific Psychopathological Forms At Department Of Youth Psychiatry, Federal State Budgetary Scientific Institution «Mental Health Research Center», Moscow, Russian Federation; 2 Researching Group Of Specific Forms Of Mental Disorders, FSBSI Mental Health Research Center, Moscow, Russian Federation

**Keywords:** end-world delusion, apocalyptic delusion, schizophrénia, psychopathology

## Abstract

**Introduction:**

Diagnostics of Apocalyptic variant of end-world Delusion with Religious Content (ADRC) in schizophrenia is related with insufficient exploration and recognizability, despite the severity of the state, social risks and resistance to psychopharmacotherapy.

**Objectives:**

To define psychopathological and phenomenological features of ADRC in schizophrenia, to identify the clinical dynamics of delusional disorders due to specifics of the delusional behavior, and to develop diagnostic and prognostic criteria.

**Methods:**

28 patients with ADRC in schizophrenia were examined (ICD-10: F20.0, F20.01, F20.02). Clinical-psychopathological and statistical methods were applied.

**Results:**

Delusional ideas of end-world, Apocalyptic variant, occurred in the structure of affective-delusional state (acute sensual delusion with fantastic content). Two types of ADRC were identified: with the predominance of acute sensory delusions of perception and with the predominance of visual-figurative delusions of the imagination. These types differed in the severity and depth of psychotic manifestations and in the specifics of a delusion formation, were characterized by the mono- or polythematic delusional disorders.

**Conclusions:**

Cases of ADRC differ both in the clinical-psychopathological specifics of delusional constructions, and in the socio-behavioral aspect. Among these cases, there is a high risk of delusional destructive behavior, with auto-aggressive, suicidal attempts and hetero-aggressive behavior. In cases with ADRC the strong persistence of delusional pseudo-religious beliefs occurs, with the refusal of any medical and psychological assistance, as well as implication of socially dangerous acts associated with the spread of delusional ideas and their induction of religiously inclined persons, which leads to the emergence of pathological pseudoreligiosity (distortion of traditional canonical religious views).

**Disclosure:**

No significant relationships.

